# Investigation of coal and gas outburst risk by microseismic monitoring

**DOI:** 10.1371/journal.pone.0216464

**Published:** 2019-05-23

**Authors:** Anhu Wang, Dazhao Song, Xueqiu He, Linming Dou, Zhenlei Li, Ziyin Zu, Quan Lou, Yingjie Zhao

**Affiliations:** 1 Laboratory of Educational Ministry for High Efficient Mining and Safety in Mental Mine, University of Science and Technology Beijing, Beijing, China; 2 School of Civil and Resources Engineering, University of Science and Technology Beijing, Beijing, China; 3 Zhong-an Academy of Safety Engineering, Beijing, China; 4 Key Laboratory of Deep Coal Resource Mining, Ministry of Education of China, School of mines, China University of Mining and Technology, Xuzhou, Jiangsu, China; 5 Guizhou panjiang refined coal co., Ltd, Panzhou, China; 6 School of Resources and Safety Engineering, China University of Mining and Technology (Beijing), Beijing, China; Beijing Forestry University, CHINA

## Abstract

In order to improve the monitoring and prediction of coal and gas outburst, this paper proposes a new method for dynamic regional prediction of coal and gas outburst using microseismic (MS) monitoring. The theoretical basis of this method is presented. An index evaluation system was established and applied, based on which field tests were carried out in a coal mine. The results show that seismic monitoring with frequency and energy indexes can obtain good results for mining disturbance intensity monitoring and geological structure detection; the regional stress distribution detected by seismic wave tomography is consistent with the theoretical stress field, making its use of great significance for optimizing coal and gas outburst drilling parameters and improving overall tunneling efficiency. This approach overcomes the limitations of traditional methods in the temporal and spatial dimensions and realizes dynamic and continuous monitoring of coal and gas outburst-prone areas.

## Introduction

Coal and gas outburst is one of the most catastrophic of coal mining hazards. This is especially so in China, where about 510 coal and gas outburst accidents occurred between 2001 and 2017, resulting in 3,576 deaths. With increasing mining depth and intensity, this hazard is becoming even more frequent [1,2]. A large amount of research and practical experience has confirmed that there are certain regularities in the occurrence of coal and gas outburst globally. The majority of coal and gas outbursts occur in geological structural zones [3,4], and their spatial distribution is controlled by three factors [5,6], namely in-situ stress, coal body structure, and gas pressure.

Many methods based on these influencing factors have been put forward for regional monitoring and early warning of coal and gas outburst. Li et al. [7] proposed a method for predicting the risk of coal and gas outburst based on spatial chaos theory using the gas desorption index of drill cuttings. Zhang et al. [8] presented an artificial neural network and fault tree analysis model based on gas content and gas pressure index. Song et al. [9] developed a regional prediction method based on multi-factor pattern recognition. Tang et al. [10] comprehensively considered the influence of the above-mentioned parameters (ground stress, gas pressure, coal strength), and proposed a line prediction technology for forecasting coal and gas outbursts. Qiu et al. [11,12] studied the variation law and precursory characteristics of EMR signals before and after coal and gas outburst.

Thus, coal and gas outburst monitoring and prediction have mainly used the gas content or gas pressure at a certain point to predict the regional risk to a coal seam, while the influence of stress change and mining disturbance have received less consideration. Additionally, the above indicators lack continuity in the temporal and spatial dimensions. In order to solve those problems, a new method is needed that is capable of real-time, continuous monitoring of coal and gas outbursts.

In recent years, there have been considerable developments in microseismic (MS) monitoring technology, and it has become a useful tool for the detection of faults, hidden cracks and stress states in underground mining [13,14]. Generalized MS monitoring is usually divided into three categories in which the scale of monitoring, main vibration frequency monitored and positioning accuracy differ. The first is large-scale monitoring, which mainly monitors large-scale rupture events with a positioning accuracy of about 500 m. It is mainly used for monitoring earthquakes [15]. The second is medium-scale monitoring. This is mainly used to monitor small-scale rupture events; the vibration frequency monitored is generally less than 150Hz, and its positioning accuracy is about 30–80 m. It has been widely used for monitoring and giving early warning of rock burst [16,17]. The last is small-scale monitoring, which mainly monitors the fracturing of the coal and rock mass in a small area (such as around a mine roadway). The vibration frequency monitored is over 300 Hz. This is usually referred to as an acoustic emission (ground sound) system [18,19].

In mines with coal and gas outbursts, a large number of studies have confirmed that small-scale MS monitoring (such as acoustic emission) has good sensitivity to the process of uniaxial, conventional triaxial and cyclic loading and unloading failure of the coal and rock mass. Regarding the application of medium-scale MS monitoring, Lei et al. [20,21] studied frequency characteristics in four phases of coal and gas outburst, i.e., incubation, excitation, occurrence, and residual, using MS methodology. Lu et al. [22] suggested that dynamic load disturbance and static stress concentration are two main factors causing coal and gas outburst and that it is feasible to use MS monitoring to assess coal and gas outburst risk. Yang et al. [23] constructed a prediction and analysis system for coal and gas outburst based on MS monitoring technology. Si et al. [24] found that there is a direct correlation between microseismicity and the gas emission rate and found that the gas emission rate tends to reach a peak at the same time as a dramatic increase in seismic energy.

The above research into the MS methodology for coal and gas outburst has mainly focused on small-scale monitoring. Medium-scale MS monitoring of coal and gas outburst has mostly been studied via theoretical analysis, and there have been few pilot applications of this technology in coal mines.

This paper is the first attempt to apply medium-scale MS technology to monitor the spatial and temporal law of MS events in mines prone to coal and gas outburst and to detect the regional stress field of outburst by using seismic wave CT. The results are used to guide the prevention and control of coal and gas outburst. Based on the experimental results, the feasibility and technical advantages of MS monitoring and early warning of coal and gas outburst are discussed.

## Regional prediction of coal and gas outburst hazard based on MS monitoring technology

### Theoretical basis

Since the first coal and gas outburst was recorded in France in 1834, a large number of coal and gas outburst accidents have occurred. The following characteristics are typical of these incidents.

The occurrence of coal and gas outburst has a clear relation to geological structure. The investigations carried out by Shepherd et al. [9] for outburst occurrences in Australia, North America, Europe, and Asia indicated that over 90% of significant outbursts were concentrated in the narrow strongly deformed zones along the axes of structures such as asymmetrical anticlines, the hinge zones of recumbent folds, and the intensely deformed zones of strike-slip, reverse and normal faults. Farmer and Pooley [25] found that coal and gas outbursts occurred only in districts that were subjected to severe tectonic movement in association with deformation and that featured certain depositional structures, in particular, rapid fluctuations in seam thickness. Cao et al. [26] investigated four coal mines in China and reported that coal and gas outbursts almost always occurred in the footwalls of reverse faults.

The occurrence of coal and gas outburst is also closely related to the degree of ground stress and mining disturbance. It is generally believed that the higher the stress around the coal seam, the greater the coal and gas outburst risk. The most direct embodiment of this is the increasing probability of coal and gas outburst at stress concentration areas, especially in the upper and lower coal pillar areas of the adjacent strata. Cheng et al. [27] pointed out that ground stress plays a leading role in coal and gas outburst disasters and is the main driving force for coal failure. The relationship between ground stress, gas pressure, and mining disturbance can be qualitatively expressed by the following equation.

$$\frac{\sigma_{1}+p+\sigma_{2}}{\sigma_{c}}\geq 1$$ (1)

where *σ*_1_ is the ground stress of the coal rock mass, *p* is the gas pressure of the coal seam, σ_2_ is the stress from mining disturbing, and *σ*_*c*_ is the minimum stress required for coal and gas outburst. Of the three factors, mining disturbance has the greatest influence on coal and gas outburst, which is reflected in the following three aspects:

1. Mining disturbance can destroy the original stress equilibrium state of a coal rock mass, causing stress to become redistributed. Consequently, a zone of increased stress forms, and the stress level of the coal seam rises.
2. The release of the stress or strain caused by the mining of coal and rock leads to deformation, fracture and even destruction of coal or rock. As a result, the strength (σ_c_) of the coal rock mass is degraded.
3. Mining disturbance greatly promotes gas desorption from coal and promotes gas flow to the excavation space. The results of Xie et al. [28] show that there is typically a coupling effect between coal seam gas pressure and mining stress and that they are positively correlated. Gas pressure reaches a peak prior to peak mining stress.

**Fig 1:**
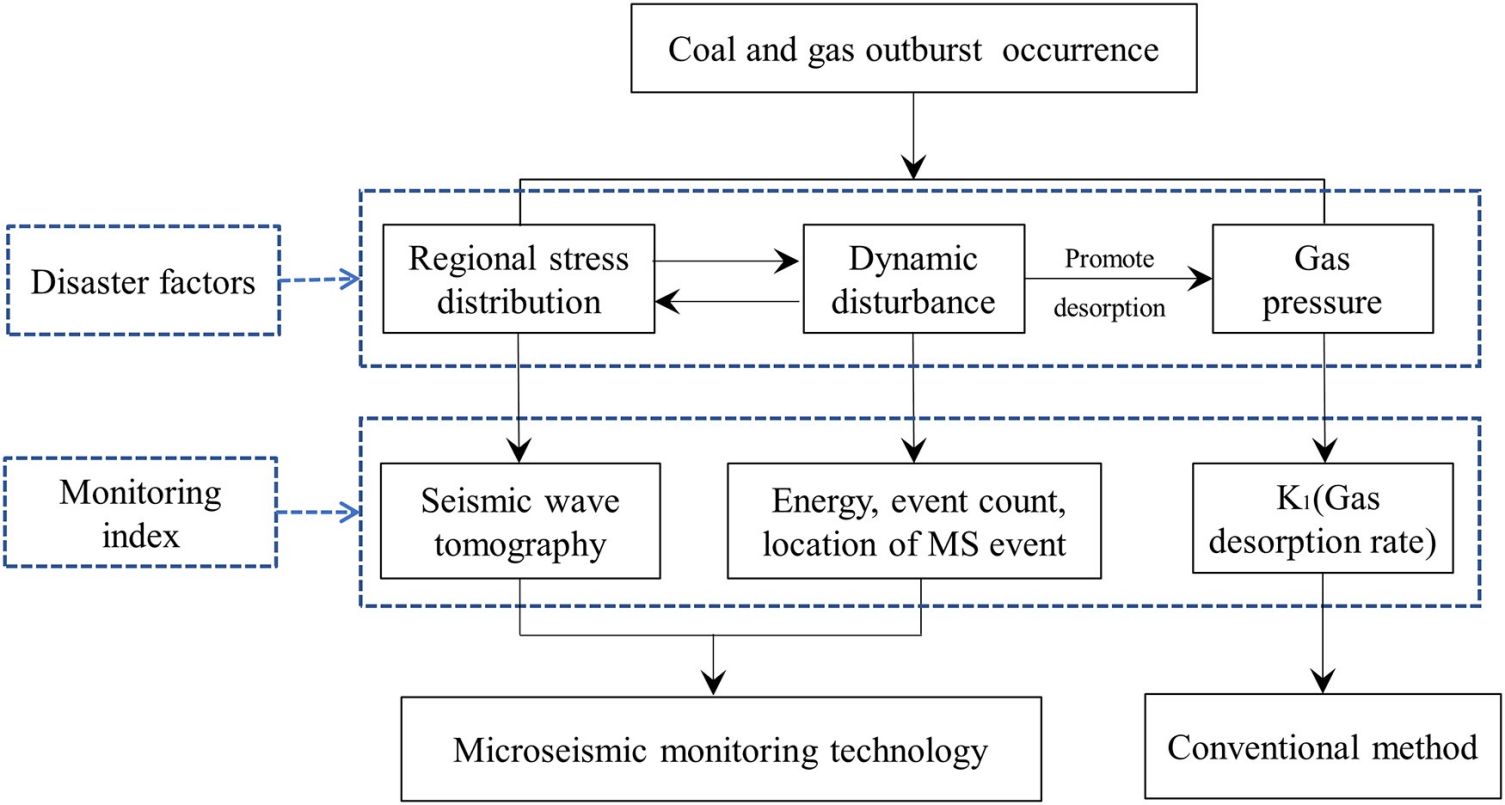
Flow diagram of MS monitoring of coal and gas outburst.

Based on the above analysis, this paper proposes a new method for the dynamic regional prediction of coal and gas outburst using MS monitoring technology, offering an effective complement to conventional prediction methods. Fig 1 presents a flow diagram of MS monitoring of coal and gas outburst.

### Technical principles

The principle of MS monitoring and positioning technology is to arrange several seismic sensors around the monitoring area, then pick up the waveform produced by seismic activity and transmitted through the coal and rock mass.

According to the arrival time (*t*_i_) of the seismic wave at each pickup sensor, the travel time residuals (Δt_i_) of the waves can be calculated using the specific model

$$\Delta t_{i}=\frac{\sqrt{{\left(x_{i}-x_{0}\right)}^{2}+{\left(y_{i}-y_{0}\right)}^{2}+{\left(z_{i}-z_{0}\right)}^{2}}}{v}-(t_{i}-t_{0})$$ (2)

where (*x*_i_, *y*_i_, *z*_i_) is the 3D coordinate of the #i pickup sensor, (*x*_0_, *y*_0_, *z*_0_, *t*_0_) is the 3D coordinate and the arrival time of the vibration; *v* is the propagation velocity of shock waves in the rock mass.

The objective function based on Δ*t*_i_ is presented below.

$$F(x_{0},y_{0},z_{0},t_{0})=\sum_{i=1}^{n}W_{i}\cdot |\Delta t_{i}{|}^{p}$$ (3)

where *W*_*i*_ is the distance weight of the pickup sensor; *P* is the standard parameter, taking the value 1 or 2.

Optimization algorithms are used to select the value for (*x*_0_, *y*_0_, *z*_0_, *t*_0_) where the target function is evaluated to a minimum. According to the power attenuation rule of the shock wave in the rock mass, the vibration energy *E* is calculated from the shock waveform using the integral method. The energy attenuation trend of the seismic wave in the rock mass decreases exponentially with an increase in propagation distance; this can be expressed as:

$$E=E_{0}L^{-\eta }$$ (4)

where *η* is the attenuation index, which has a value related to the propagation medium.

Friedel et al. [29,30] found that the high wave velocity region has a good correspondence with the stress concentration area, and the longitudinal wave velocity and stress magnitude have an obvious power function relationship. Therefore, the regional stress field can be evaluated on the basis of the distribution of longitudinal wave velocity values.

This evaluation can be implemented via the following steps: According to the distance *L* between the sensor and the shock location and the time (*T*) at which the sensor picks up the vibration signal, the wave velocity distribution *V(x*, *y*, *z) (unit*: *m·s*^*-1*^*)* or slow degree *S (x*, *y*, *z) = 1/V (x*, *y*, *z)* (unit: s·m^-1^) is calculated. We describe the distance between the sensor and the shock location as *L*_i_ and the pickup time as *T*_i_. The relationship between *L*_i_ and *T*_i_ can be represented by the following equations.

$$\begin{array}{cc} V=L/T & L=VT \end{array}$$ (5)

$$T_{i}=\int_{L_{i}}\frac{dL}{v(x,y,z)}=\int_{L_{i}}s(x,y,z)dL$$ (6)

$$T_{i}=\sum_{j=1}^{M}d_{ij}\cdot S_{j}(i=1,\ldots ,n)$$ (7)

where *d*_*ij*_ is the length of the *i*th shock wave ray passing through the *j*th grid; *N* is the total number of rays; *M* is the number of grids.

The matrix form of the above relationship is as follows.

$$\begin{array}{cc} T=DS & S=D^{-1}T \end{array}$$ (8)

where *T* is the column vector of the shock wave (N×1); *S* is the slowness column vector (M×1); *D* is the ray length matrix.

**Fig 2:**
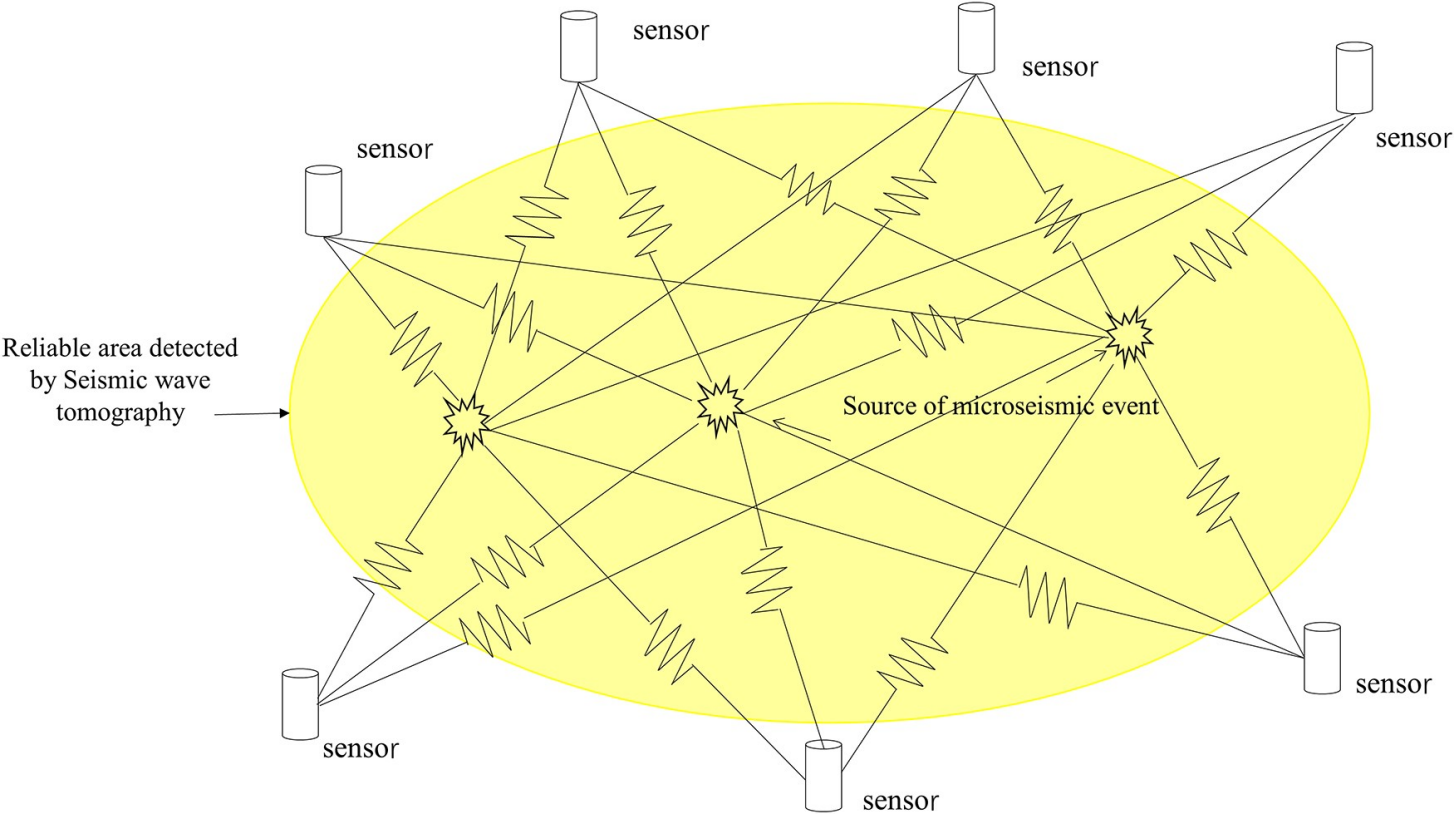
Detection technology principles of seismic wave tomography.

In general, Eq (8) is an over-determined or undetermined system of equation and needs to be solved iteratively. Because using the simultaneous iterative reconstruction technique (SIRT) for the iterative algorithm does not have singular solutions and can converge smoothly, this paper uses the SIRT algorithm to perform the operation. Fig 2 illustrates the detection technology used for seismic wave tomography.

### Index system

MS monitoring and positioning technology mainly uses energy and frequency indicators. These two indicators have universal applicability to the study of regional MS activity. The energy indicator represents the intensity of the energy released by MS activity, which is the key indicator with which to determine the magnitude of the MS event. The frequency indicator reflects the number of MS events per unit time, which, in combination with the spatial distribution characteristics of vibration events, can reflect the degree of concentration of shock location.

In order to detect the relative size of the regional stress field at a coal seam, the wave velocity anomaly coefficient (*A*_n_) is calculated on the basis of the average wave velocity.

$$A_{n}=\frac{v_{p}-v_{p}^{a}}{v_{p}^{a}}$$ (9)

where $v_{p}^{a}$ is the average model wave velocity.

According to formula (9), when velocity *v*_*p*_ is greater than the average model wave velocity $v_{p}^{a}$, the wave velocity anomaly coefficient *A*_*n*_ is a positive number, which means that stress is relatively high in this area. Conversely, when the velocity *v*_*p*_ is less than the average velocity $v_{p}^{a}$, the velocity anomaly coefficient *A*_*n*_ is a negative number, meaning that the stress is relatively low in this area.

Table 1 shows the relationship between abnormal wave velocity and the degree of stress concentration. Table 2 shows the relationship between negative wave speed anomalies and the degree of stress reduction.

**Table 1:** Relationship between positive wave speed anomalies and stress concentration.

concentration characteristic	positive wave speed anomaly *A*_*n*_*/*%	probability of stress concentration
none	<5	<0.2
weak	5~15	0.2~0.6
medium	15~25	0.6~1.4
strong	>25	>1.4

**Table 2:** Relationship between negative wave speed anomalies and stress reduction.

reduction characteristic	negative wave speed anomaly *A*_n_/%	probability of stress reduction
none	0~-7.5	<0.25
weak	-7.5~-15	0.25~0.55
medium	-15~-25	0.55~0.8
strong	<-25	>0.8

## Field application

### Mine geology and workface layout

**Fig 3:**
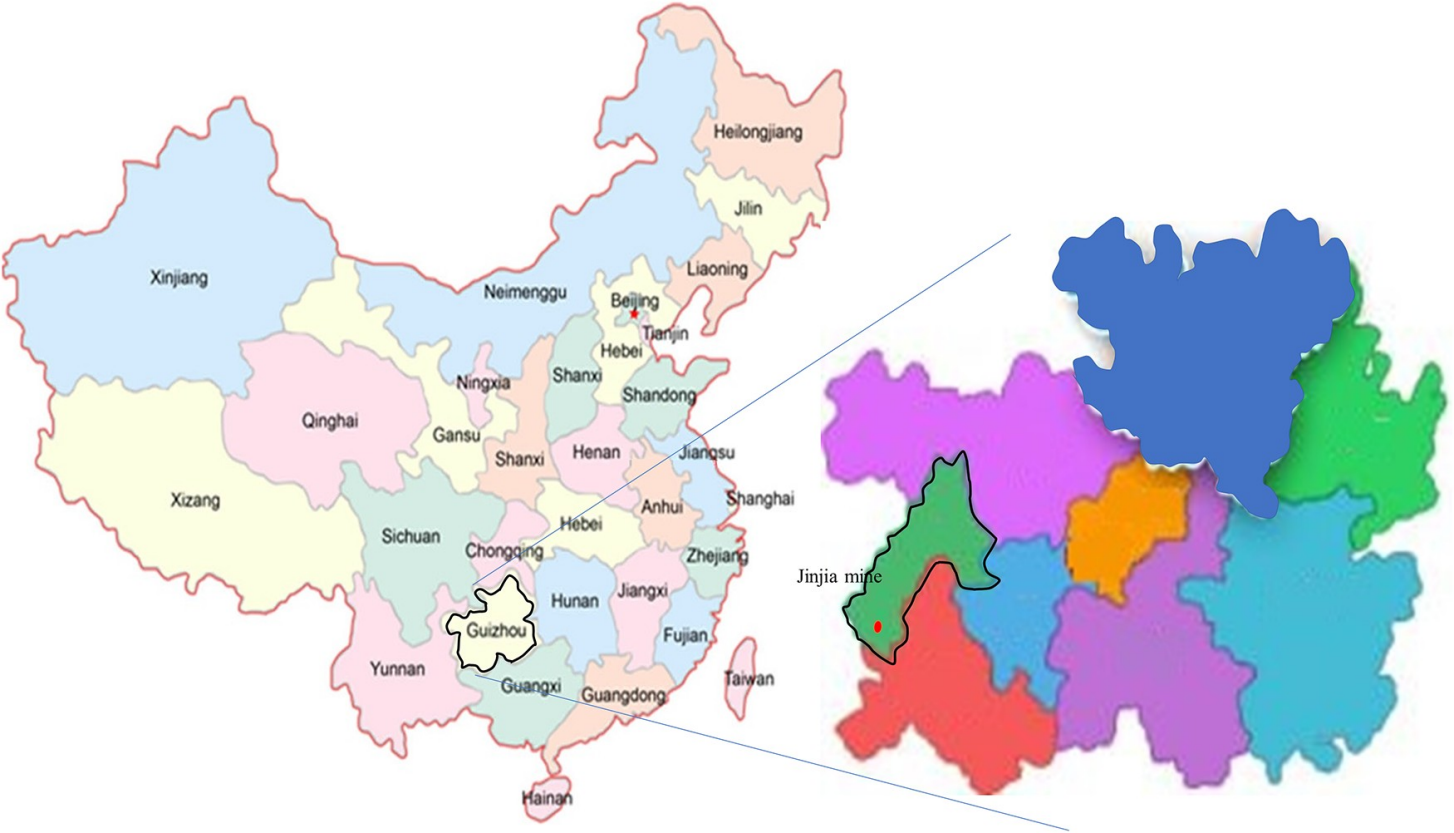
Location of Jinjia coal mine, Liupanshui city, Guizhou province, China.

The experiment was carried out in Jinjia Coal Mine, located in Liupanshui City, Guizhou Province, China. The location of the mine is shown in Fig 3. It is a typical coal and gas outburst-prone mine and has a production capacity of 1.8 million t/a. As shown in Table 3, at least two coal and gas outbursts causing serious casualties and property loss have occurred since it began production.

**Table 3:** Detailed description of coal and gas outbursts in Jinjia Mine.

Time	Location	Coal quantity	Casualty	Property loss (RMB)
2007.6.7	1175 transport lane	10t	4 deaths	No record
2013.1.18	211 cross cut	No record	13 deaths	17.05 million

The key area for this test is at working face No. 11224, located in the first area mined. The design length of the haulage roadway is 438 m, and that of the open-off cut is 180 m. There is a geological anomaly approximately 30 m to the right of the open-off cutting face.

The parameters of the coal seam are as follows. It is 1.2 m thick and has a dip angle of 22°, the gas content of the original coal seam is 10.8 m^3^/t, the firmness coefficient *f* of the coal seam is 0.5–0.6, and the burial depth of the workface is 100–400 m. According to the results of coal and gas outburst hazard identification by China University of Mining and Technology, No. 22 coal seam is prone to coal and gas outburst.

**Fig 4:**
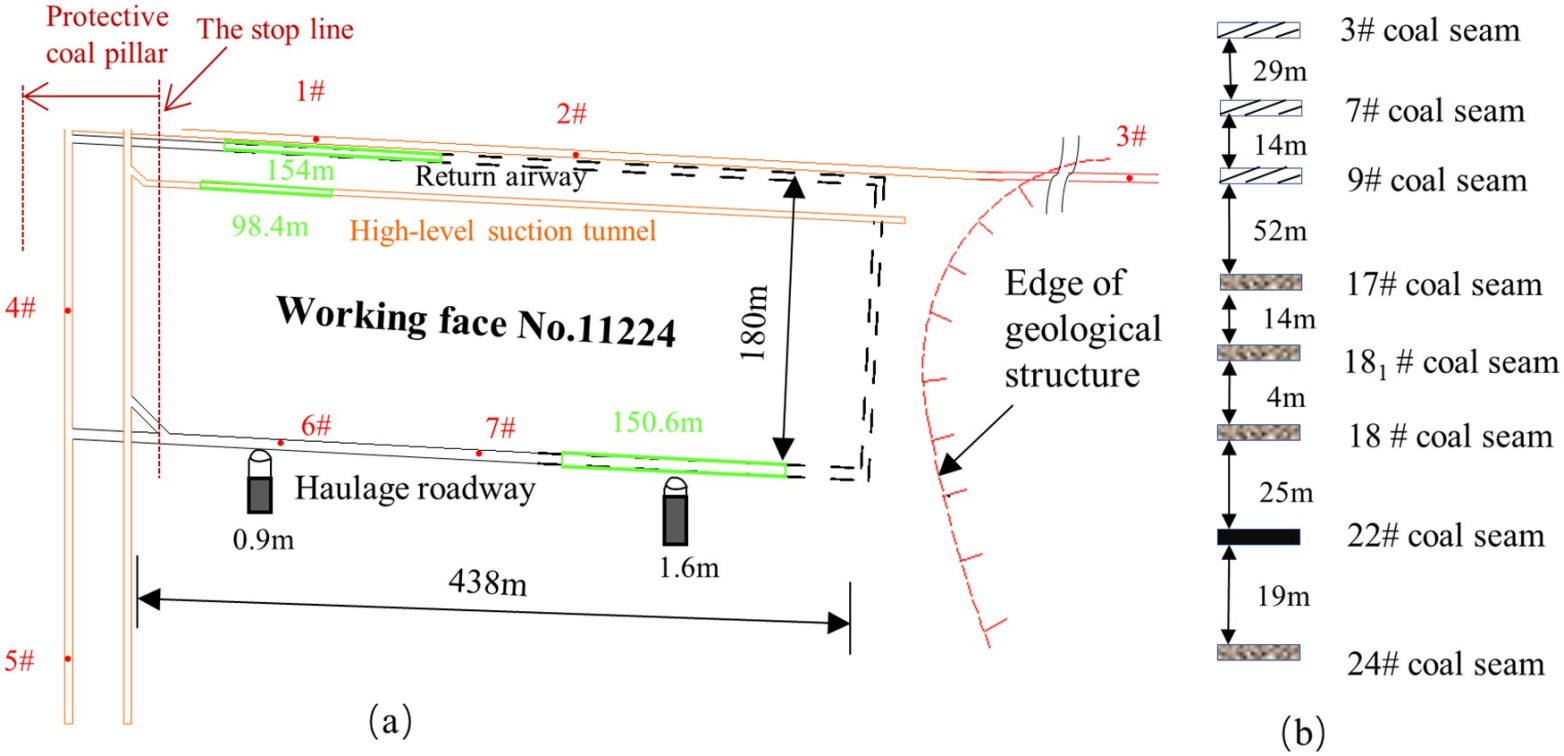
(a) Working face No. 11224 and the location of MS sensors; (b) the coal seams 3#, 7#and 9# had been mined above working face No. 11224; the 22# coal seam was mainly monitored for this paper; the coal seams 17#, 18_1_#, 18# and 24# were original unexploited. The red dots represent the locations of the sensors.

MS events in the working area were monitored from June 18th,2017 to October 10th,2017. During this period, the haulage roadway was actually driving 150.6 m, the return airway was actually driving 154 m, and the high-level suction tunnel was actually driving 98.4 m; the green lines in Fig 4(a) indicate these sections.

The main measures to prevent coal and gas outburst in the tunneling working face are implemented pre-extraction. Gas extraction is performed 120 m in front of the tunneling working face in each cycle. After the extraction has qualified as complete, an additional 100 m driving along the coal seam laneway is allowed per cycle in theory. However, during the monitoring period from June 18th to October 10th, the haulage roadway was only driven 55.6 m from July 1st to July 14th, 32 m from August 4th to August 11th, and 63 m from September 14th to September 25^th^; these interruptions were all due to an increased risk of coal and gas outburst.

### Setup of MS monitoring system

In order to explore the results of coal and gas outburst monitoring, the new generation SOS MS monitoring system developed by the Polish Mine Research Institute was installed in the mine in June 2017. This system can sense MS events within a maximum scope of 13 km in real time. Combined with the roadway deployment surrounding working face No. 11224, a total of 7 MS sensors were arranged surrounding the roadway, which constitutes effective coverage of the working face No. 11224 area. The layout of the MS sensors is shown in Fig 4(a).

### Spatial and temporal distribution of MS signals

#### Spatial distribution of MS signals

**Fig 5:**
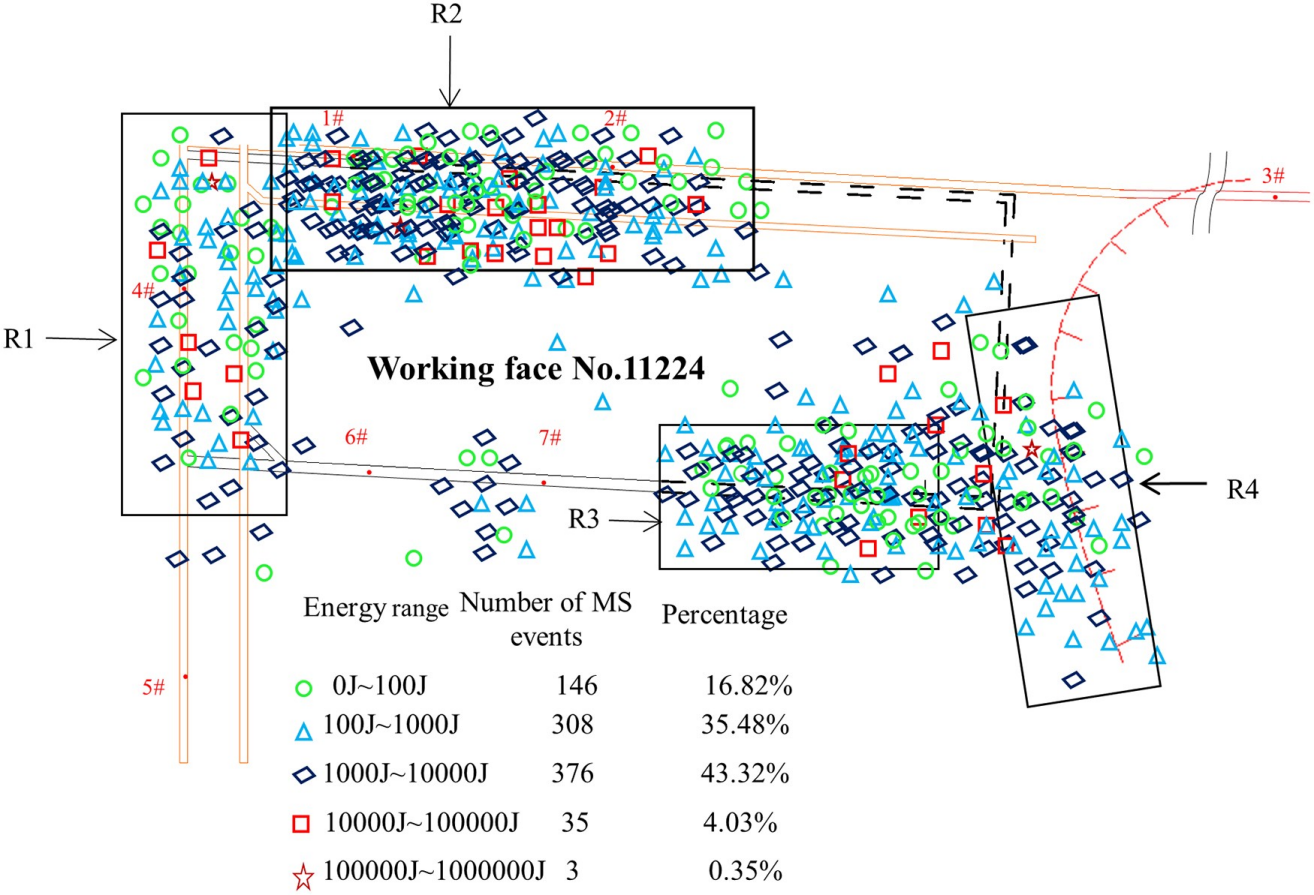
The planar distribution of MS events.

A total of 868 MS signals were detected in the test area between June 18 and October 10, of which only three MS events were of a magnitude over 10^5^J. However, low-energy incidents below 10^3^J accounted for 52.3%, and events in the range 10^3^J –10^5^J accounted for as much as 47.35%. The spatial distribution of the MS signals is shown in Fig 5.

The above image shows that the MS signals were mainly concentrated in regions R1–R4. An analysis of the reasons for this shows a strong correlation with the following factors.

**Fig 6:**
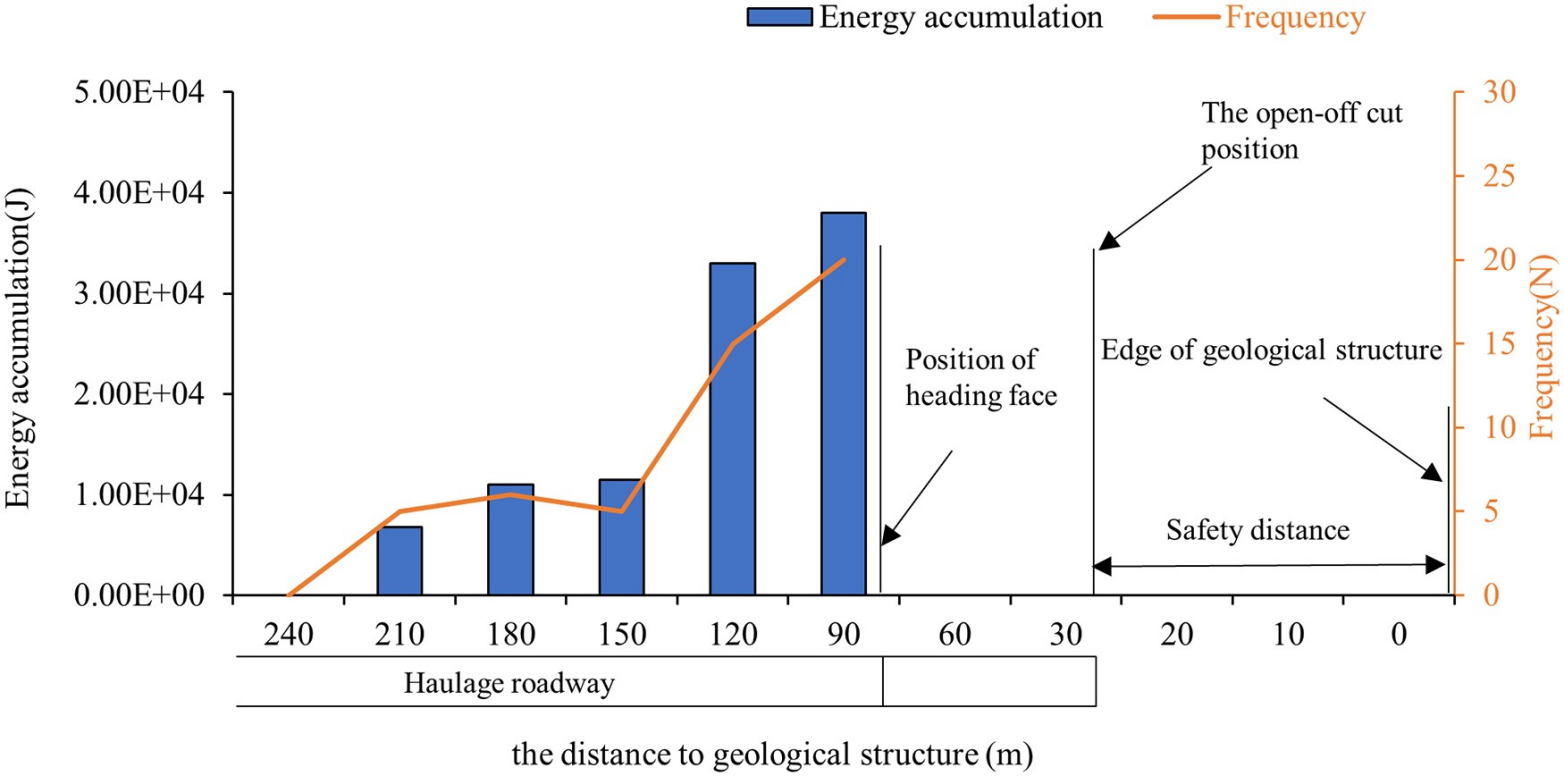
Accumulated energy and frequency of MS events from the heading face to the geological structure. The above results indicate that the distribution characteristics of the MS events are closely related to mining activities and anomalous geological bodies.

1. R1 region. The MS events in this area should be mainly caused by the protective coal pillars in the mining area.
2. R2 region. This area is influenced by the return airway and high-level suction tunnel. As a result, the disturbance of the blasting face is greatest here, and the number of MS signals detected is the highest.
3. R3 region. This area is the zone disturbed by the haulage roadway; the MS signals in this region have obvious timing characteristics, a specific analysis of which is reported in Section 3.3.2.
4. R4 region. This is close to a geological structure that is located approximately 30 m outside the position of open-off cutting. The frequency of mine earthquakes and energy release in this area are closely related to the distance between the heading and the geological structure. As shown in Fig 6, the frequency and energy of MS events in the region R4 begin to increase dramatically when the heading head is 120 m away from the geological structure.

#### Temporal law of MS signals

Region R3 is the main disturbance region of the coal heading during this time, and the frequency and intensity of disturbance have a major influence on the risk of coal and gas outburst.

**Fig 7:**
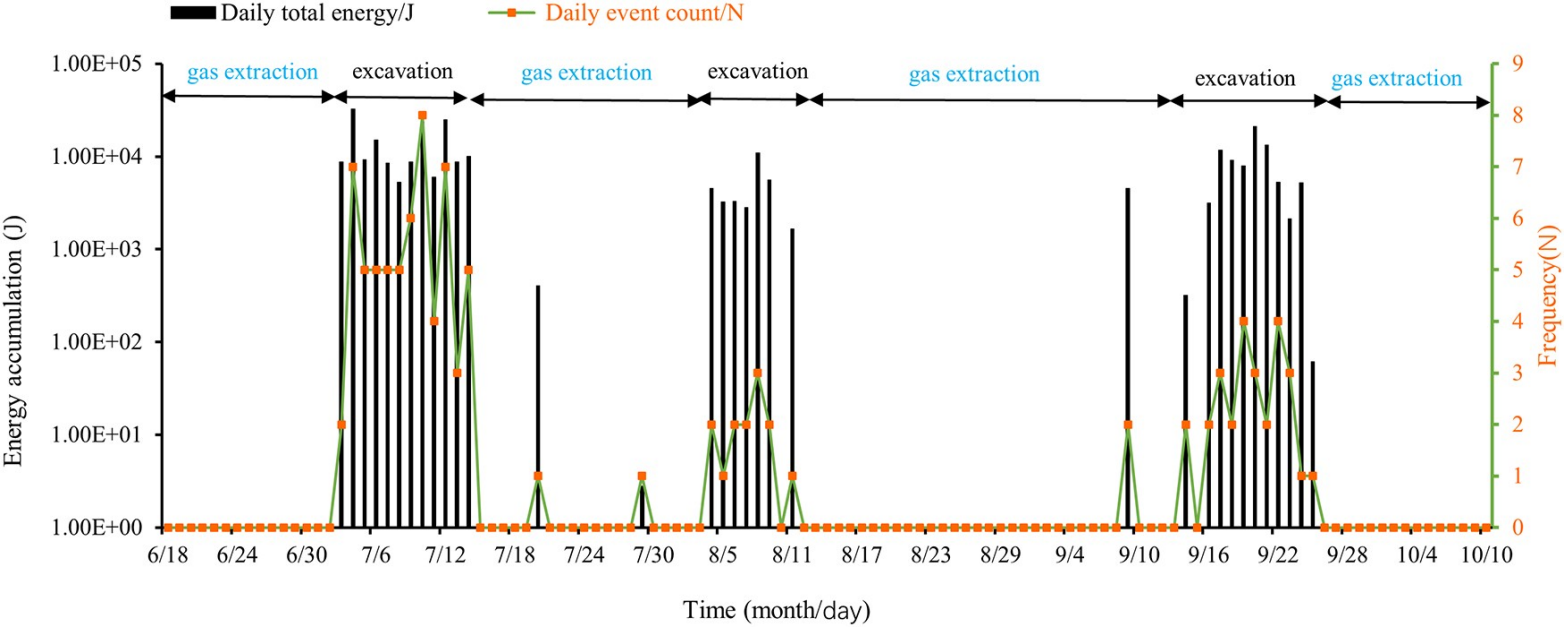
Timing of changes in MS signals around the heading area.

The cumulative frequency and energy of the daily MS signals in the vicinity of region R3 were calculated from June 18 to October 10, and their timing was compared with that of the roadway excavation or gas extraction process. A sequence diagram of the MS signals was obtained and is shown in Fig 7.

It can be clearly seen in Fig 7 that the frequency of occurrence and release energy of MS signals near the heading face of the coal seam roadway were closely related to the working procedure at the roadway. During the roadway tunneling process, the MS energy and event count increased significantly in concert with major mining disturbances. When the workface driving stopped, the frequency index and release energy index of the MS signals around the workface became relatively low.

**Fig 8:**
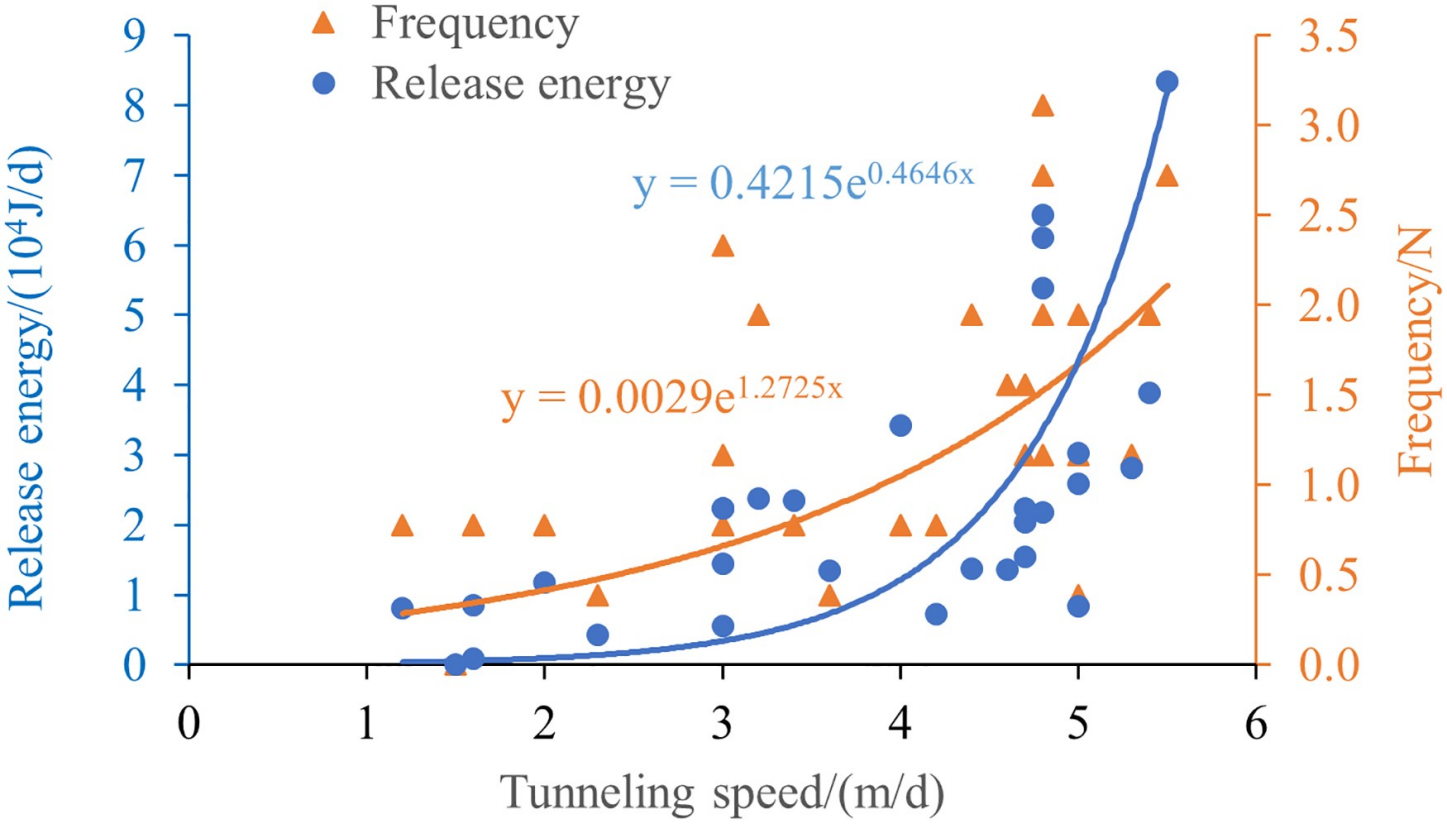
The relation between MS frequency, release energy and tunneling speed.

In addition, in order to further study quantitative relationship between MS frequency, energy and tunneling speed, the daily tunneling length is regarded as the tunneling speed (m/d). Therefore, the daily excavation lengths of 7/05-7/14, 8/04-8/11, 9/16-9/25 excavation periods are counted, and the quantitative relationship between MS frequency, release energy and tunneling speed are obtained as shown in Fig 8.

From Fig 8, it can be seen that the tunneling speed is between 1.2m/d and 5.8m/d, the MS frequency and release energy increase exponentially with increase of tunneling speed. This verifies that the frequency of occurrence and release energy of MS events around the workface are positively correlated with the degree of mining disturbance. Therefore, it can be concluded that the degree of disturbance of coal seam mining can be monitored effectively by using the MS monitoring frequency index and energy index.

### Results of seismic wave tomography

**Fig 9:**
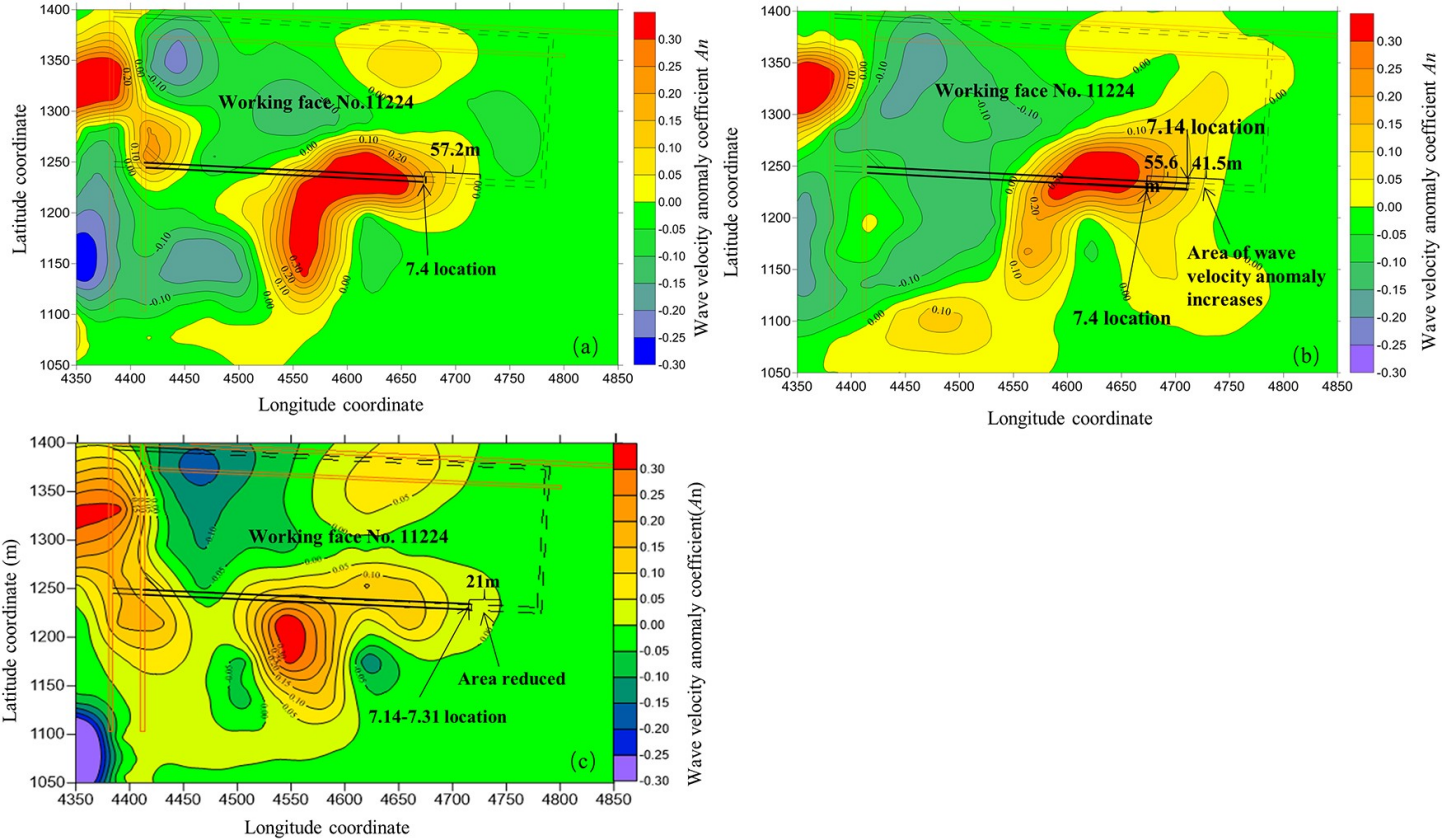
(a) Contour map of the wave velocity anomaly coefficient *A*_n_ in the 6/15–7/04 gas drainage period; (b) Contour map of the wave velocity anomaly coefficient *A*_n_ in the 7/05–7/14 roadway excavation period; (c) Contour map of the wave velocity anomaly coefficient *A*_n_ in the 7/15–7/31 gas drainage period.

This study investigates the distribution of the regional stress field along with mining drivage by using seismic wave tomography to detect the dynamic evolution of the regional stress field of the coal and gas outburst-prone coal seam. Fig 9 presents the results respectively detected in three stages: 6/18/2017 to 7/04/2017 (gas extraction), 07/05/2017 to 7/14/2017 (roadway excavation), and 7/15/2017 to 7/31/2017 (gas extraction). The wave velocity anomaly coefficient *A*_*n*_ at an elevation of +1670 m, the same elevation as the haulage roadway, is selected as the detection and evaluation result of the stress field around working face No. 11224.

The analysis in Fig 9 shows that the regional stress field at working face No. 11224 experienced dynamic change with the advance of tunneling. Also, the position of the stress anomaly was strongly consistent with the actual excavation process. The influence of tunneling work and gas extraction on the stress field around the head is analyzed as follows:

1. The coal pillar area is located at the left side of working face No. 11224, where the wave velocity anomaly coefficient (*A*_n_) is relatively high, consistent with the theoretical position of stress concentration.
2. Comparing the test results in Fig 9(b) with those in Fig 9(a) shows that, with the advance of the roadway heading, the wave velocity anomaly coefficient (*A*_n_) in Fig 9(b) increased compared with the value at the same position in Fig 9(a). This is because the area of concentrated stress in front of the working face moves forward along the roadway and is also consistent with the increasing risk of coal and gas outburst in front of the working face.
3. Comparing the test results in Fig 9(c) with those in Fig 9(b) shows that, with the increase in gas extraction in front of the working face, the extent of the area with a positive wave velocity anomaly reduced from 41.5 m in Fig 9(b) to 21 m in Fig 9(c). This is consistent with a reduction in stress and gas content through the drilling of a large number of holes in front of the working face, gradually causing the risk of coal and gas outburst to decrease.

The above results show that the regional stress distribution detected by seismic wave tomography is consistent with the theoretical stress field in an outburst-prone coal seam. This technology can dynamically and exactly detect the characteristics of the evolution of the stress field caused by mining activities such as coal roadway excavation and gas extraction.

### Application and verification

#### MS monitoring response to geological structures and its verification

**Fig 10:**
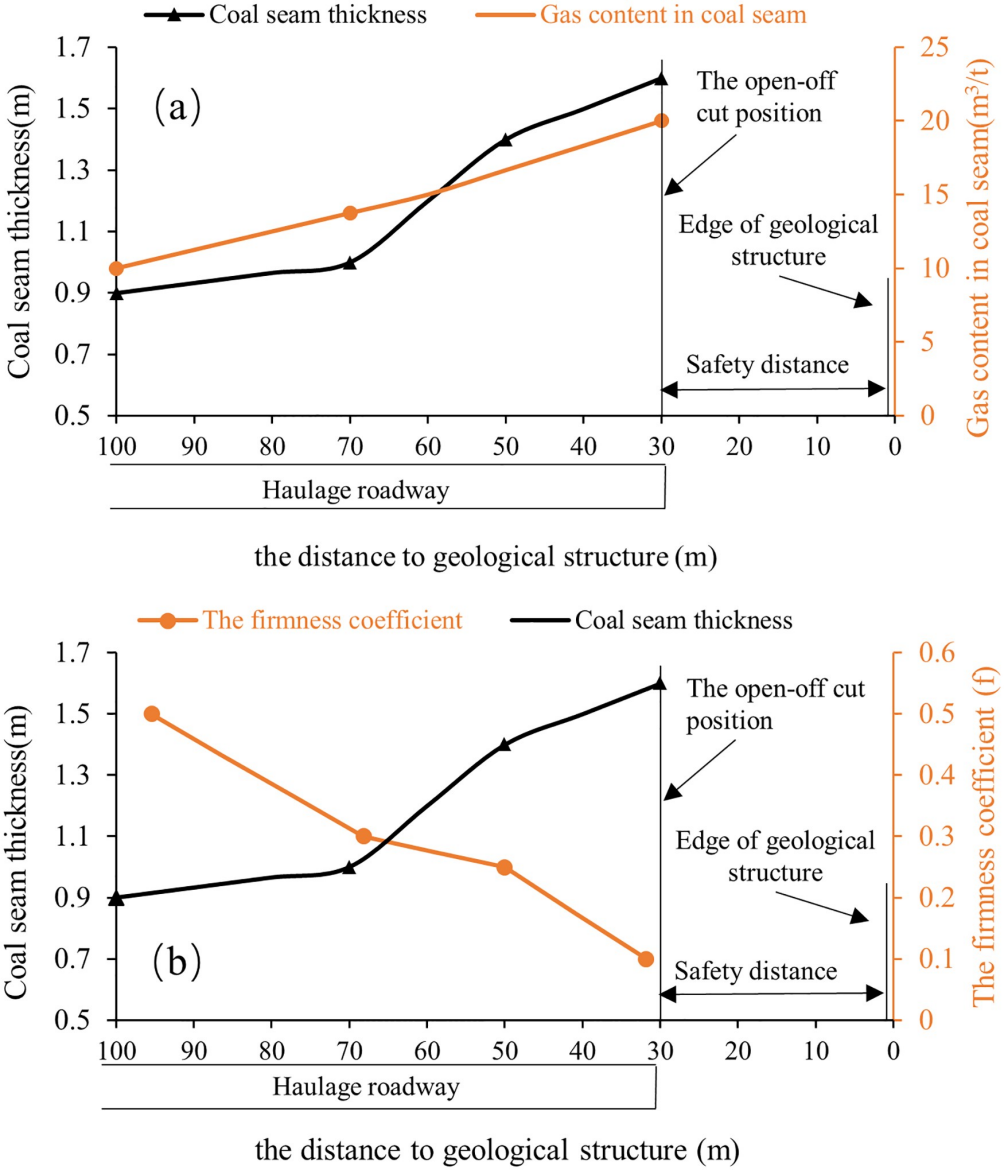
(a) Variation in coal seam thickness and gas content with distance to a geological structure; (b) Variation in the coal seam thickness and firmness coefficient *f* with distance to a geological structure.

A basic feature of geological structures is that they lead to obvious changes in coal seam occurrence parameters. According to the statistics of coal seam parameters along the haulage roadway, as shown in Fig 10, from 100 m away from the location of the theoretical geological structure to the cutting position of working face, the coal seam thickness rises from 0.9 m to 1.6 m, the measured gas content rises from 10 m^3^/t to 20 m^3^/t, and the firmness coefficient *f* decreases from 0.5 to 0.1. The above parameters are sufficient to confirm the existence of a geological structure.

By comparing and analyzing the results in Section 3.3.1 and Fig 6, the distance from the roadway heading to the theoretical geological structure is inferred to be about 120 m, and there is an obvious increase in the frequency and energy of MS events in region R4.

These phenomena fully prove that the MS monitoring technique can satisfactorily detect geological structures in advance in a coal and gas outburst-prone mine.

#### Verification of regional stress field detection via electromagnetic radiation(EME)

A large number of research results [31,32,33] have shown that the intensity of EME can reflect the stress state of a coal and rock mass. When the stress is high, there is a large amount of deformation and damage in the coal body, and the intensity of EME is high.

**Fig 11:**
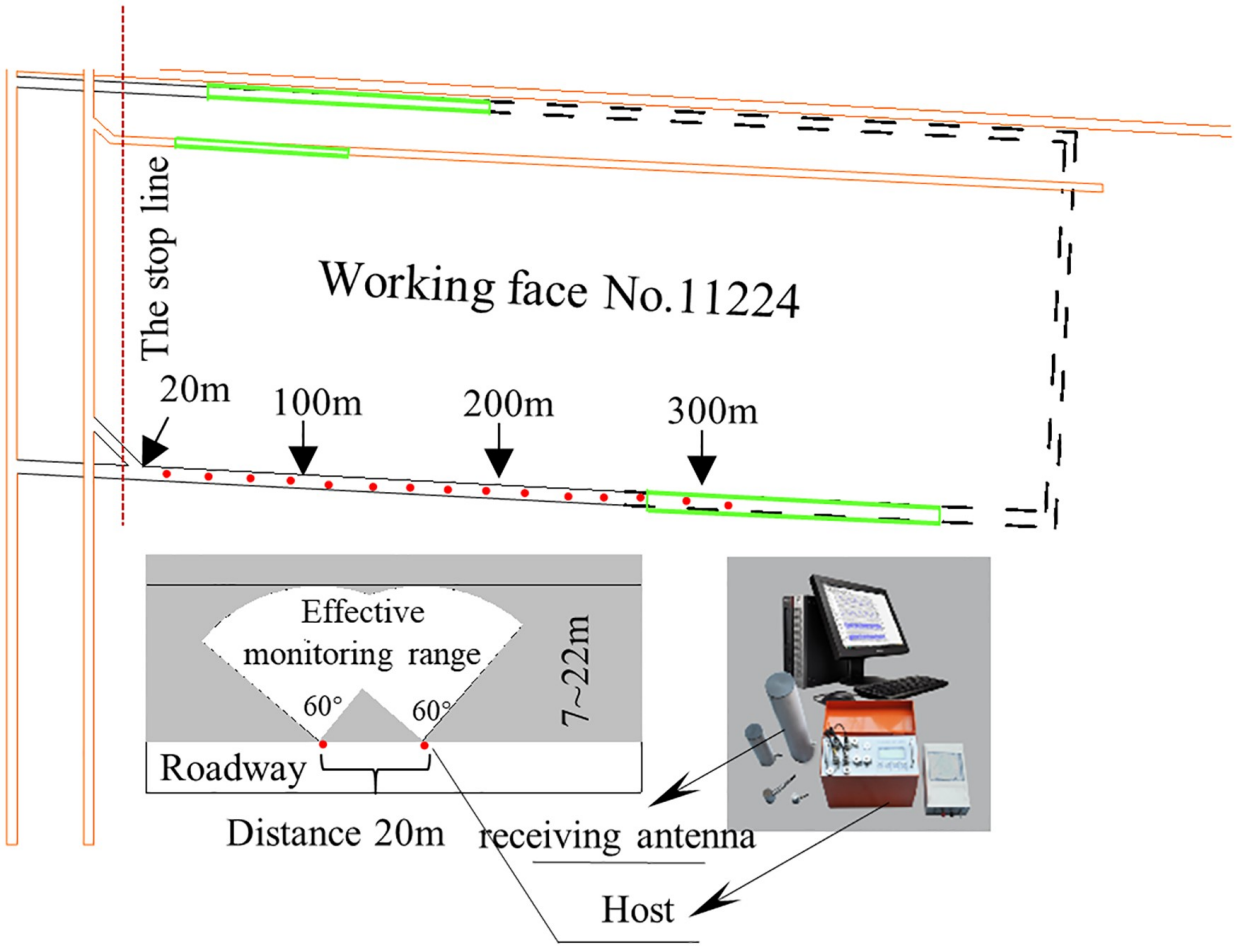
Monitoring principles and scheme of the KBD5 monitor.

**Fig 12:**
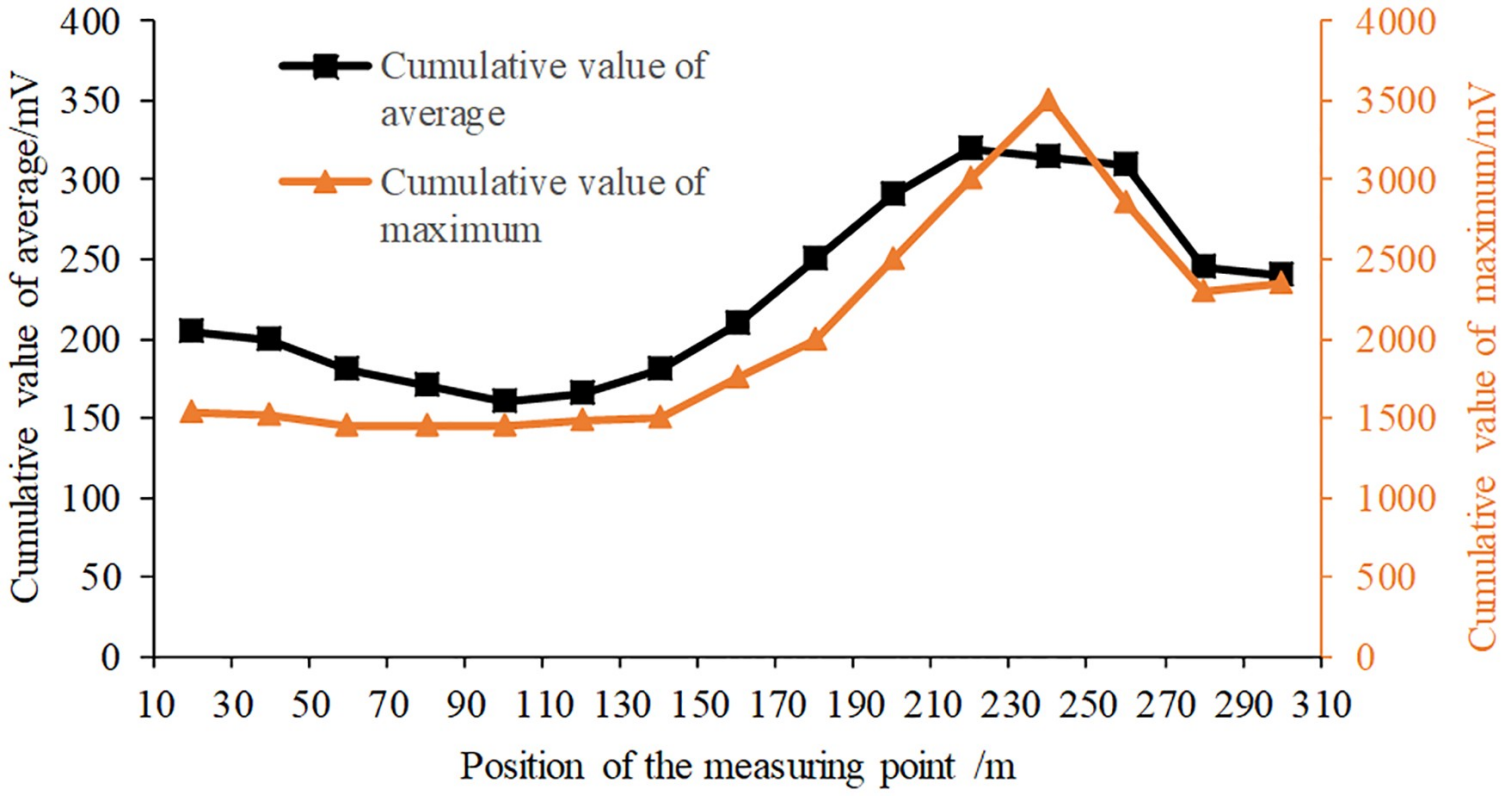
Monitoring results from the KBD5 monitor.

In order to verify the accuracy of seismic wave CT in detecting the distribution of the regional stress field in a coal seam, EME test data from July 25 to August 3, 2017 were selected for analysis. Fig 11 shows the principles and scheme for monitoring EME in haulage roadway No. 11224. Fig 12 shows the average sum and maximum sum curves of EME intensity at each monitoring point during the monitoring period.

As can be seen from Fig 12, the EME monitoring results correspond well with the inverted wave velocity inversion results in this period (shown in Fig 9). That is, the intensity of EME is higher in the high wave velocity region, whereas the intensity of EME is lower in the low wave velocity region.

## Discussion

### Comparison of MS monitoring and traditional prediction indexes of coal and gas outburst

Analyzing the field test results from MS monitoring in Jinjia mine indicates that MS technology is capable of effectively detecting the distribution of regional stress in a coal and gas outburst-prone coal seam. At the same time, it can dynamically monitor the intensity of mining disturbance and the distribution of geological structures. Deep analysis of MS region prediction results in Jinjia mine, compared with traditional coal and gas outburst monitoring and forecasting methods such as drill cutting ΔH_2,_ K_1,_ q, artificial neural network and fault tree analysis models, multi-factor pattern recognition, etc., MS monitoring technology has conspicuous advantages.

1. Continuous monitoring. Artificial neural network and fault tree analysis models, multi-factor pattern recognition, traditional coal and gas outburst monitoring and forecasting methods, etc. all using coal seam property parameters or gas parameters to evaluate the risk of coal seam outburst. The gas desorption indexes (i.e., ΔH_2,_ K_1,_ q) are used to evaluate the risk of coal and gas outburst. None of these can realize continuous monitoring. As shown in Table 4 below, it is the result of predicting the risk of coal and gas outburst by K_1_ value method during period of 7/8/2017 to1/20/2018 in Jinjia Mine. The prediction of K_1_ value method is measured once every 7 meters, which can not realize continuous monitoring. However, MS monitoring can realize continuous monitoring of mining disturbance information and regional stress field, which can be used to predict the risk of coal and gas outburst.
2. Regional dynamic monitoring. Unlike with the traditional monitoring and prediction methods, MS monitoring technology realizes continuous dynamic exploration of the stress field and geological structure in outburst-prone coal seams. These factors are the key to prediction of coal and gas outburst occurrence. By the end of February 2019, the MS monitoring system of outburst risk in Jinjia mine has been continuously monitoring for nearly 20 months.
3. Accurate location of the position of the disturbance source. Dynamic load disturbance and static stress concentration are two of the main factors in coal and gas outburst. Without mining disturbance, coal and gas outburst accidents will not occur. Therefore, dynamic monitoring of mining disturbance and its intensity is of great significance for monitoring and giving early warning of coal and gas outburst.
4. Ascertaining the area of stress concentration. As discussed in Section 3.4, the stress distribution in the coal seam obtained by seismic wave tomography technology is basically the same as the theoretical distribution.

**Table 4:** The date and result of K_1_ value determination in Jinjia Mine.

Date	The result of K_1_ value	Date	The result of K_1_ value
7/8/2017	0.16	10/26/2017	0.18
7/9/2017	0.17	10/27/2017	0.18
7/10/2017	0.18	10/28/2017	0.17
7/12/2017	0.14	10/31/2017	0.18
7/13/2017	0.15	11/3/2017	0.16
7/14/2017	0.38	11/5/2017	0.17
8/4/2017	0.24	11/6/2017	0.16
8/6/2017	0.16	12/1/2017	0.09
8/8/2017	0.27	12/4/2017	0.2
8/9/2017	0.27	12/5/2017	0.18
8/11/2017	0.25	12/7/2017	0.17
8/13/2017	0.28	12/8/2017	0.15
8/15/2017	0.52	12/10/2017	0.52
9/17/2017	0.22	1/7/2018	0.18
9/19/2017	0.26	1/9/2018	0.18
9/20/2017	0.26	1/11/2018	0.18
9/22/2017	0.25	1/13/2018	0.17
9/23/2017	0.22	1/15/2018	0.18
9/24/2017	0.14	1/18/2018	0.23
10/25/2017	0.18	1/20/2018	0.31

Although MS technology has conspicuous advantages for monitoring coal and gas outburst, is also has the following shortcomings:

1. The results for the regional stress field detected by seismic wave tomography are only relative: the absolute stress value in the region is not obtained. Therefore, using seismic wave tomography to determine the degree of coal and gas outburst danger has some limitations. However, it is undeniable that the distribution of the regional relative stress field can determine the relative magnitude of coal and gas outburst risk in different positions, and this is of great significance for identifying the key danger areas so as to prevent and control coal and gas outburst.
2. Both coal and gas outburst and rock outburst are typical dynamic coal-rock disasters in coal mines, and their occurrence mechanism and characteristics have many similarities. MS monitoring technology has been successfully applied many times in the field of rock burst monitoring and early warning. However, the coal seam and its roof or floor are usually stronger in a rock outburst-prone seam than in a coal and gas outburst-prone seam. This is because the energy release in a rock outburst-prone seam is larger than in a coal and gas outburst-prone seam. Therefore, the accuracy of early warnings of coal and gas outburst from MS monitoring technology should be further discussed.

### Exploration of seismic wave tomography and roadway tunneling length

**Fig 13:**
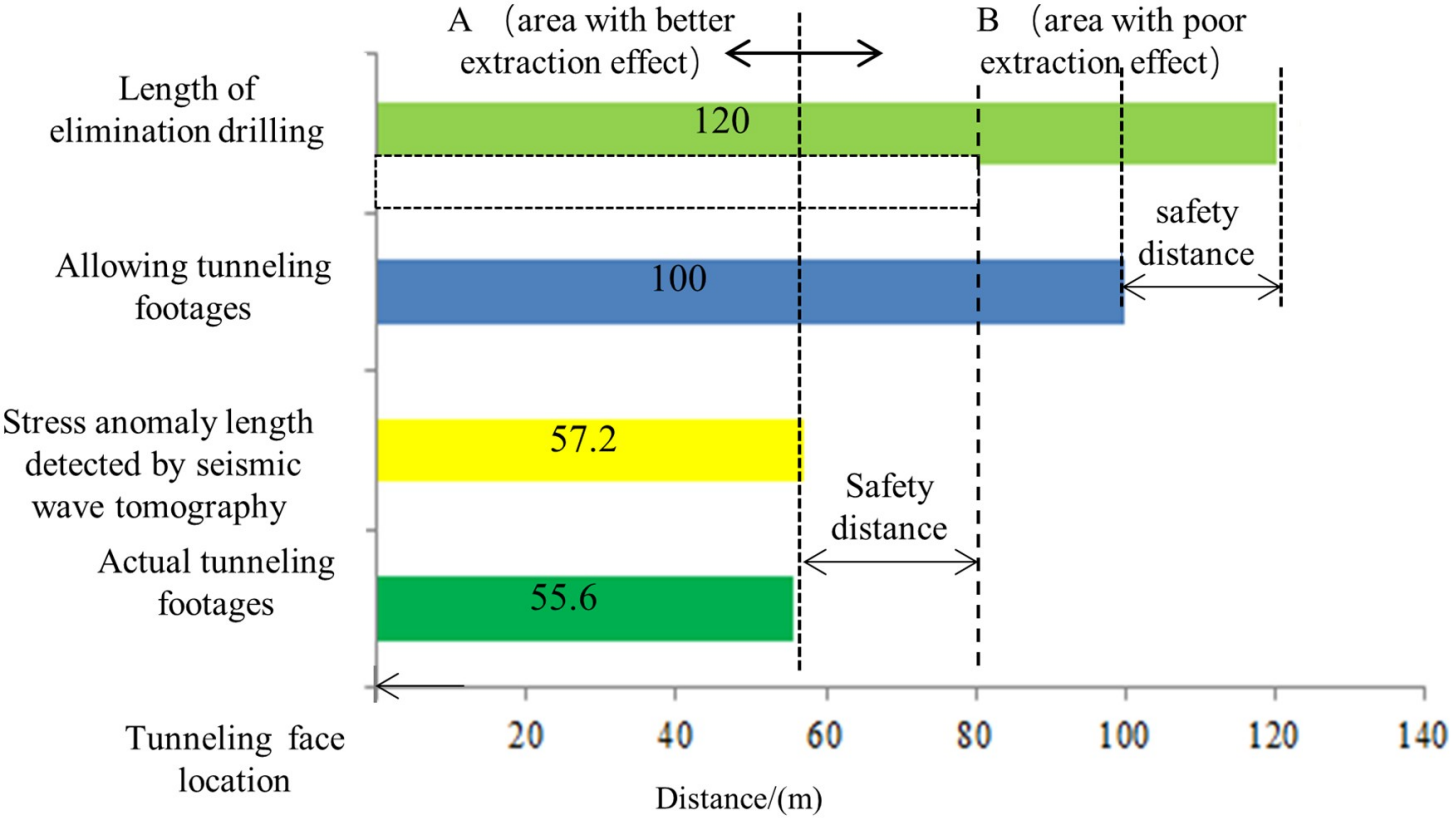
Summary of roadway excavation information.

In the period 6/18/2017 to 07/04/2017 (gas extraction), the layout length of the gas extraction borehole was 120 m. After gas elimination reached the standard level, the allowed tunneling footages are 100 m after subtraction of the 20 m safety distance. However, the extent of the stress anomaly in front of the tunneling head detected by seismic wave tomography was 57.2 m (Fig 9(a)) and the actual tunneling footages were only 55.6 m (Fig 9(b)) because K_1_ (a conventional index for the prediction of coal and gas outburst) is over-limiting. This is illustrated in Fig 13.

The actual tunneling length is a good reflection of the elimination effect. From Fig 13, we can see that the effect of gas extraction in area A is better than that in area B.

Based on the above analysis, it’s possible that seismic wave tomography can be applied to optimize the layout parameters of extraction drilling, reduce the quantity of construction engineering, and improve the efficiency of roadway driving. For example, on the premise of meeting the safe distance, the drilling depth can be reduced from 120 m to 80 m. In this way, the length of the borehole can be reduced by one third. In other words, the drilling time could be shortened and the tunneling efficiency could be improved. Therefore, seismic wave tomography may be of great significance for optimizing the layout parameters of coal and gas outburst drilling and improving the overall roadway tunneling efficiency. Further research into this would therefore be worthwhile.

## Conclusion

Through theoretical research and analysis of field application, we can draw the following conclusions:

1. MS monitoring technology can achieve dynamic monitoring and regional stress field detection of coal seam mining disturbance and geological structure. Its application shows that the geological structure and regional stress field detected by MS monitoring technology are consistent with those predicted theoretically, which verifies the feasibility of this technology for the regional prediction and early warning of coal and gas outburst as an effective complement to conventional prediction methods.
2. MS monitoring technology overcomes the limitations of conventional regional prediction in time and space and achieves dynamic and continuous monitoring of coal and gas outburst-prone coal seams.
3. It is of great significance to determine the key areas for coal and gas control by early detection of the regional stress field distribution of the coal seam, geological structure, and so on.
4. Seismic wave tomography may be of great significance for optimizing the layout parameters of coal and gas outburst drilling and improving overall roadway tunneling efficiency. Further research on this topic would be valuable.

## List of symbols

**Table:** 

*σ*_1_	The ground stress of the coal rock mass
*p*	The gas pressure of the coal seam
*σ*_2_	The stress from mining disturbing
*σ*_*c*_	The minimum stress required for coal and gas outburst
*t*_i_	The waves arrival time
Δ*t*_i_	The travel time residuals of the waves
(*x*_i_, *y*_i_, *z*_i_)	The 3D coordinate of the #i pickup sensor
(x_0_, y_0_, z_0_, t_0_)	The 3D coordinate and the arrival time of the vibration
v	The propagation velocity of shock waves in the rock mass
Wi	The distance weight of the pickup sensor
P	The standard parameter, taking the value 1 or 2
E	The vibration energy
dij	The length of the ith shock wave ray passing through the jth grid
N	The total number of rays
M	The number of grids
T	The column vector of the shock wave (N×1)
S	The slowness column vector (M×1)
D	The ray length matrix
$v_{p}^{a}$	The average model wave velocity
An	The wave velocity anomaly coefficient
Vp	The wave velocity
f	The coal seam thickness and firmness coefficient

## Supporting information

S1 File Data for Figs 5–8 and 12. (XLSX)
